# Intra-abdominal desmoid tumor mimicking gastric cancer recurrence: a case report

**DOI:** 10.1186/1477-7819-12-146

**Published:** 2014-05-10

**Authors:** Akihiko Okamura, Tsunehiro Takahashi, Yoshiro Saikawa, Shuhei Mayanagi, Kazumasa Fukuda, Rieko Nakamura, Norihito Wada, Hirofumi Kawakubo, Tai Omori, Hiroya Takeuchi, Aya Sasaki, Yuko Kitagawa

**Affiliations:** 1 Department of Surgery, School of Medicine, Keio University, 35 Shinanomachi, Shinjuku-ku, Tokyo 160-8582, Japan; 2 Division of Surgical Pathology, Keio University Hospital, 35 Shinanomachi, Shinjuku-ku, Tokyo 160-8582, Japan

**Keywords:** Desmoid tumor, Gastric cancer, Recurrence, Surgery

## Abstract

Intra-abdominal desmoid tumors are rare and most often occur in patients with a history of familial adenomatous polyposis, surgery, or pregnancy. We report a case of an intra-abdominal desmoid tumor mimicking the recurrence of gastric cancer. A 57-year-old male had undergone distal gastrectomy for advanced gastric cancer. Serum levels of carcinoembryonic antigen were found to be elevated 27 months after surgery. Computed tomography revealed a 15-mm mass in the mesentery of the transverse colon. In addition, radiotracer fluorodeoxyglucose uptake of the tumor was detected by positron emission tomography. The patient was diagnosed with gastric cancer recurrence, and chemotherapy consisting of cisplatin and S-1 was commenced. After five courses of chemotherapy, although no significant clinical response was seen, no new lesions were seen either. Thus, a curative resection of the recurrent tumor seemed possible, which was successfully performed. Histological examination of the resected specimen revealed spindle-shaped tumor cells with collagen fiber progression; no cancer cells were detected. The tumor was diagnosed as an intra-abdominal desmoid tumor. We report a rare case of an intra-abdominal desmoid tumor that mimicked a recurrent tumor arising from gastric cancer. In patients with history of surgery for intra-abdominal malignancies, it may be difficult to distinguish the recurrence of malignancy from desmoid tumors but the possibility of desmoid tumors must be considered in the differential diagnosis.

## Background

Desmoid tumors are rare connective tissue tumors that occur most often in patients with a history of familial adenomatous polyposis (FAP), surgery, or pregnancy [1]. Although they are histologically benign and non metastatic, they are locally invasive. Few reports exist on intra-abdominal desmoid tumor that mimics gastric cancer recurrence after gastrectomy [2-4]. In this report, we report a rare case of an intra-abdominal desmoid tumor that mimicked a recurrent tumor arising from gastric cancer.

## Case presentation

**Figure 1:**
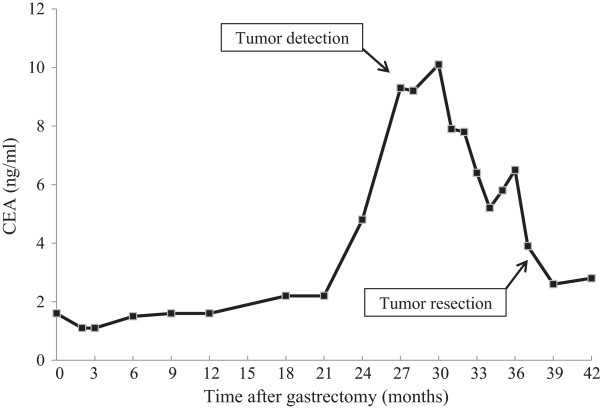
**The changes in serum carcinoembryonic antigen level after gastrectomy. **CEA, carcinoembryonic antigen.

**Figure 2:**
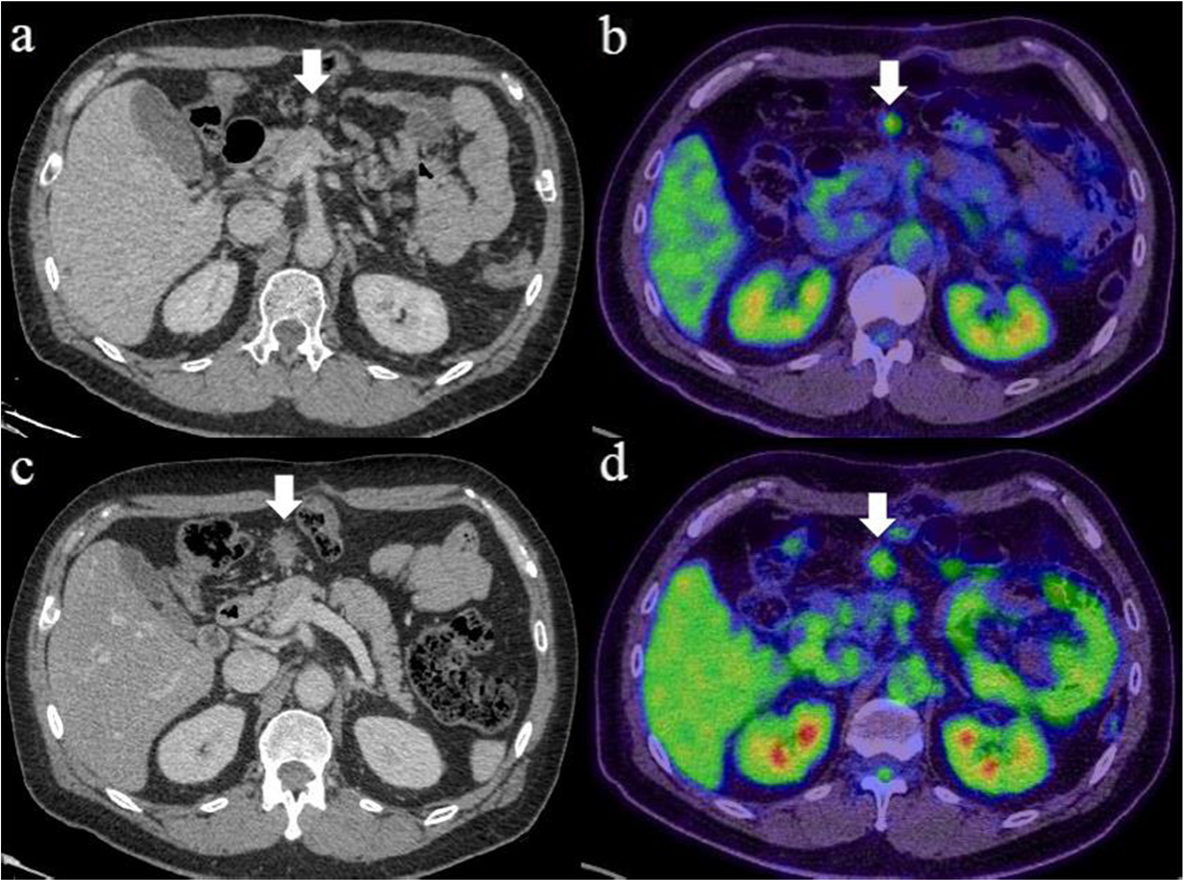
**Abdominal computed tomography (CT) and positron emission tomography (PET) of the desmoid tumor. (a) **CT shows a solitary and localized tumor in the mesentery of the transverse colon 27 months after gastrectomy (arrow). **(b) **PET shows radiotracer fluorodeoxyglucose uptake of the tumor (arrow). **(c) **CT and **(d)** PET of the tumor after five courses of chemotherapy, showing that no significant clinical response was seen (arrow).

A 57-year-old male patient had undergone distal gastrectomy and D2 lymphadenectomy for advanced gastric cancer. A postoperative abdominal abscess presented around the pancreatic head, which was improved by conservative therapy. Histopathological examination of the tumor revealed a poorly differentiated adenocarcinoma; the tumor was classified as stage IB (pT2N0M0) according to the 7th TNM classification of the International Union Against Cancer [5]. Because a curative resection was performed, adjuvant chemotherapy was not administered (according to the Japanese gastric cancer treatment guidelines 2010 [6]). His follow up for recurrence included analysis of serum carcinoembryonic antigen (CEA) level every 3 months and computed tomography (CT) and/or ultrasonography every 6 months. At 27 months after gastrectomy, an increase in serum CEA level was detected during follow up (Figure 1). CT and positron emission tomography (PET) were subsequently performed. CT revealed a solitary, localized mass measuring 15 mm in diameter in the mesentery of the transverse colon (Figure 2a). Radiotracer fluorodeoxyglucose (FDG) uptake of this tumor was seen (Figure 2b) during the PET study. The maximum standard uptake value (SUV max) of the early phase was 3.86, and that of the late phase was 2.87.

We considered that this tumor was a recurrence, in the lymph node or peritoneum, of the previous gastric cancer. We initiated first-line chemotherapy consisting of cisplatin and S-1 for five cycles. Serum CEA levels slightly decreased (Figure 1). However, no clinical response was observed in image inspections (Figure 2c, d); the tumor was noted to have slightly enlarged, but no new lesions were observed. Because curative resection seemed possible, we decided to completely resect the tumor. The tumor strongly adhered to the transverse colon; therefore, this part of the transverse colon was also removed.

**Figure 3:**
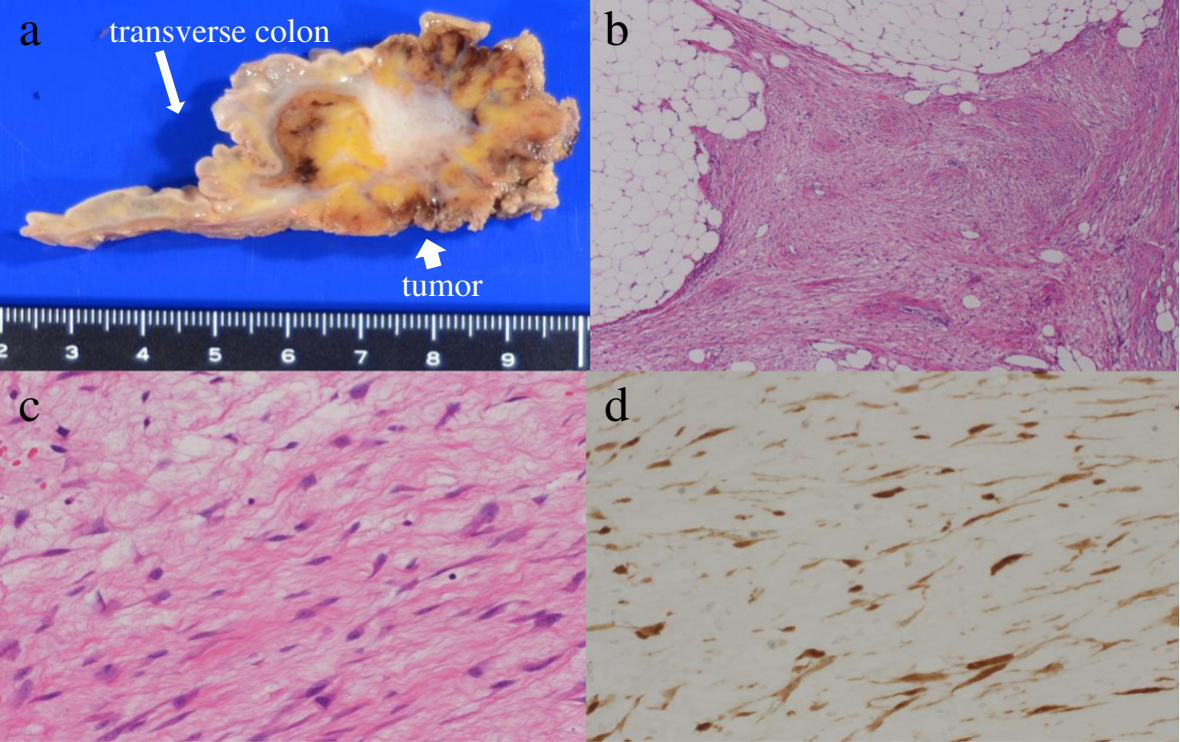
**Pathologic features of the desmoid tumor. (a)** Macroscopically, a 40 × 30 × 30 mm hard elastic tumor was seen in the mesentery of the transverse colon. The cut surface is whitish and poorly circumscribed with surrounding adipose tissue. **(b, c)** Histologically, proliferation of spindle-shaped cells with collagenous stroma is seen (hematoxylin and eosin staining; **b**: low-power field, **c**: high-power field). **(d)** Immunohistological examination revealed that the cell nuclei were positive for beta-catenin.

Macroscopically, the tumor was hard and elastic, measuring 40 × 30 × 30 mm. The cut surface was whitish and poorly circumscribed with surrounding adipose tissue (Figure 3a). Histological examination revealed proliferation of spindle-shaped cells and collagenous stroma (Figure 3b, c). No nuclear hyperchromasia or atypia was observed in these cells. Immunohistological examination revealed that the cells were focally positive for α-smooth muscle actin, negative for keratin, and nuclei-positive for beta-catenin (Figure 3d). The tumor was diagnosed as desmoid-type fibromatosis, also known as a desmoid tumor. No cancer cells were detected in the resected specimen. The patient’s postoperative course was favorable, and there was no evidence of recurrence of gastric cancer and desmoid tumor after 6 months.

## Discussion

Desmoid tumors are rare connective tissue tumors involving the muscloaponeurotic tissues; they are benign and non metastatic but locally invasive and tend to recur. They account for 0.03% of all newly diagnosed neoplasms [7] and 3% of all soft tissue neoplasms [8,9]. The etiology of desmoid tumors, which are predominantly found in young female patients of reproductive age, is unknown. However, they are strongly associated with endocrine factors, FAP, and trauma (including surgical incision) [1]. In this case, there is no family history of FAP. In addition, the patient had no colonic lesion evident on colonoscopy, which was performed routinely as a preoperative inspection. Therefore, our case was not associated with FAP. Although there are no reports of correlation between desmoid tumors and the infectious complications of previous surgery, it is possible that the etiology in our case could be related to intra-abdominal infection rather than surgical incision.

Intra-abdominal desmoid tumors mimicking gastric cancer recurrence after gastrectomy are very rare, and few cases have been reported [2-4]. In the case presented here, on identifying a solitary mass in the mesentery of the transverse colon, we initially thought it to be tumor recurrence (such as recurrent lymph node metastasis or peritoneal dissemination) - the diagnosis of desmoid tumor was never considered. In relation to other malignancies such as colorectal and renal cancer [10-12], there are also only a few cases of intra-abdominal desmoid tumors mimicking tumor recurrence after previous surgery. Therefore, postoperative intra-abdominal desmoid tumors in patients with history of surgery for intra-abdominal malignancies are not well known. However, despite an extremely low incidence, it is clear that postoperative desmoid tumors should be included in the differential diagnoses.

The tumor marker CEA is widely used as an indicator of disease progression or recurrence after resection of various malignancies. CEA is expressed in some tumors of epithelial origin, including those of the lung, ovary, breast, and colorectum; it is also sometimes expressed in normal tissue [13]. There has been no definitive study investigating serum CEA level in relation to desmoid tumors; in addition, serum CEA elevation in patients with desmoid tumors does not seem to be recognized, because they are not tumors of epithelial origin. Although serum CEA was elevated in our patient, the degree of elevation did not correlate with the tumor size in image inspections. In addition, we could not find another lesion that was thought to be a gastric cancer recurrence, or other malignancies on CT and PET. Serum CEA elevation has been associated with several non neoplastic conditions, including chronic inflammatory disease, renal and hepatic insufficiency, aging, and smoking [14]. The cause of serum CEA elevation in our patient is uncertain, but it might be related to a non neoplastic condition.

Diagnostic imaging for tumor assessment includes the use of CT, magnetic resonance imaging (MRI), and PET. Desmoid tumors are often inhomogenously enhanced on contrast CT, hypo-intense on T1-weighted MRI, and mixed but predominantly hyper-intense on T2-weighted MRI, reflecting both the proliferation of fibroblasts and increase of collagen fibers [15,16]. We did not perform MRI in our patient because we initially believed the tumor to be a recurrence. However, desmoid tumors do not show specific findings in diagnostic imaging, and few cases have been definitively diagnosed before surgical resection. PET is an imaging modality that has recently began to play an important role in the diagnosis and staging of various malignancies [17]. SUV is a semiquantitative analysis of the degree of metabolic activity in the abnormal tissues. In general, desmoid tumors seem to be detected with low FDG uptake, because of their low malignancy. However, there has been no definitive study investigating the pattern of FDG uptake and the possible clinical use of PET in diagnosing desmoid tumors; only a preliminary report exists [18]. Therefore, the clinical diagnosis of desmoid tumor can be difficult and can only be established by histological examination or molecular analysis.

As a treatment strategy, surgical resection using wide negative margins remains the first-line treatment of intra-abdominal desmoid tumor [19]; conservative management strategies for inoperable desmoid tumors remain unclear. Therefore, it is necessary to detect the tumor early and to perform appropriate surgical resection in patients presenting with desmoid tumor. However, in the case of recurrence from previous malignancies, appropriate treatment should also be performed depending on the type of malignancy and the situation. Although it is difficult to distinguish the recurrence of a malignancy from a desmoid tumor, it is important to consider the possibility of an intra-abdominal desmoid tumor mimicking tumor recurrence when performing a postoperative workup for intra-abdominal malignancy. If desmoid tumor is even slightly suspected, surgical resection should be considered.

## Conclusion

This case presents a rare case of intra-abdominal desmoid tumor mimicking gastric cancer recurrence after gastrectomy. In patients with a history of surgery for intra-abdominal malignancies, the possibility of postoperative desmoid tumor should be considered, even though distinguishing malignant recurrences from desmoid tumors remains difficult.

## Consent

Written informed consent for publication of this case report and any accompanying images was obtained from the patient. A copy of the written consent is available for review by the Editor-in-Chief of this journal.

## Abbreviations

CEA: carcinoembryonic antigen; CT: computed tomography; FAP: familial adenomatous polyposis; FDG: fluorodeoxyglucose; MRI: magnetic resonance imaging.PET, positron emission tomography; SUV: standard uptake value.

## Competing interests

The authors declare that they have no competing interests.

## Authors’ contributions

All authors except for AS were involved in the care of the patient. AS carried out the pathological studies and provided the histological figures. All authors have read and approved the final manuscript.
